# Evaluation of Richmond Agitation Sedation Scale According To Alveolar Concentration of Sevoflurane During a Sedation With Sevoflurane in Icu Patients

**DOI:** 10.1186/2197-425X-3-S1-A27

**Published:** 2015-10-01

**Authors:** S Perbet, C Fernandez-Canal, B Pereira, JM Cardot, JE Bazin, JM Constantin

**Affiliations:** CHU Clermont Ferrand, Réanimation Adultes, Pôle Médecine Péri-Opératoire, Clermont-Ferrand, France; CHU Clermont Ferrand, Réanimation Adultes, Clermont-Ferrand, France; CHU Clermont Ferrand, Biostatistics Unit, Clermont-Ferrand, France; Université d'Auvergne, Pharmacologie, Clermont-Ferrand, France

## Introduction

In ICU sedation, use of inhalated gaz has interesting qualities, but remains limited due to lack of available and appropriate material. a new medical device (Mirus^TM^ (Pall)) allows feedback of the delivered flow rate from the targeted minimal alveolar concentration (MAC). Sedation is usually monitored by the RASS (Richmond Assessment Sedation Score).

## Objectives

The objective of this study was to describe the values of RASS corresponding to different MAC or sevoflurane expired fraction (FeSevo) in sedated patients.

## Methods

This prospective, interventional study was approved by the appropriate Institutional Review Board (Comité de Protection des Personnes Sud-Est VI, Clermont-Ferrand, France; NCT02202720) and conducted in a 16-bed ICU. For each patient, the sevoflurane MAC were increased to 0.1 MAC every 30 minutes from MAC 0 until MAC 0.8 then reduced with the same steps. Sevoflurane was administered with the Mirus^TM^ system. RASS was evaluated after 15 min of stability after changing the MAC. Statistical analysis was performed using STATA, p < 0.05 was considered significant.

## Results

Thirty patients were included: 11 women and 19 men, median age was 57 years; 63% of patients were postoperatively, median IGSII was 29.7 and SOFA 5.6. Compared to MAC 0, RASS decreased significantly from MAC 0.1 (p < 0.001) then stabilized from MAC 0.6. From MAC 0.2, a significant correlation was noted between Bispectral index (BIS) and sevoflurane dose, and between the BIS and RASS. For a target of RASS -5, effective concentrations (EC) of sevoflurane were MAC 0.6 for EC50 and MAC 0.8 for EC95.

**Figure 1:**
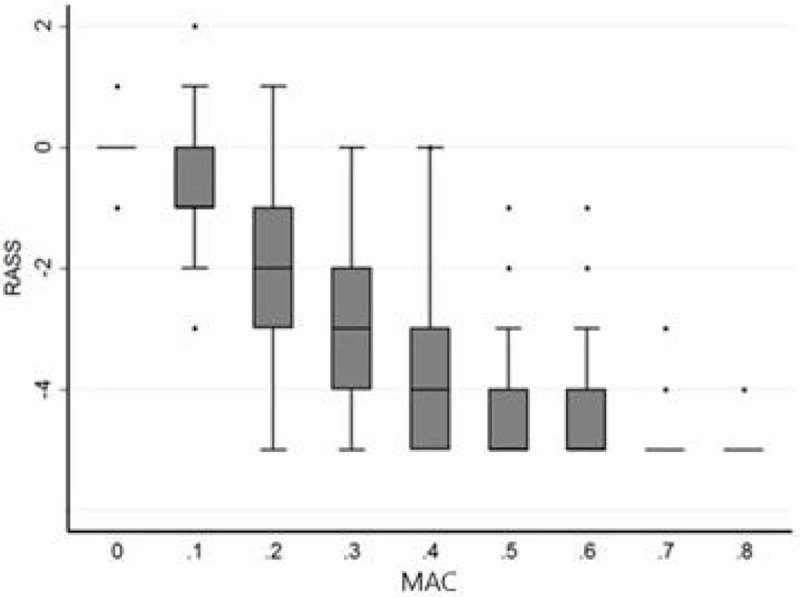
**RASS & sevoflurane MAC.**

## Conclusions

This study found a correlation between the RASS and also the FeSevo or MAC of sevoflurane. The decrease in the RASS was well correlated with the increase in the concentration of sevoflurane and decreased BIS. Sedation with inhalated sevoflurane administered by the Mirus^TM^ system allows stable concentrations of sevoflurane and was well correlated to the targeted level of sedation. EC50 and EC95 can be expressed from RASS -1 until RASS -5.

